# Steps toward broad-spectrum therapeutics: discovering virulence-associated genes present in diverse human pathogens

**DOI:** 10.1186/1471-2164-10-501

**Published:** 2009-10-29

**Authors:** Chris J Stubben, Melanie L Duffield, Ian A Cooper, Donna C Ford, Jason D Gans, Andrey V Karlyshev, Bryan Lingard, Petra CF Oyston, Anna de Rochefort, Jian Song, Brendan W Wren, Rick W Titball, Murray Wolinsky

**Affiliations:** 1 Bioscience Division, Los Alamos National Laboratory, Los Alamos, NM; 2 Biomedical Sciences, Dstl, Porton Down, Salisbury, UK; 3 School of Life Sciences, Kingston University, Kingston-upon-Thames, Surrey, UK; 4 Department of Infectious and Tropical Diseases, London School of Hygiene and Tropical Medicine, London, UK; 5 School of Biosciences, University of Exeter, Stocker Road, Exeter, UK

## Abstract

**Background:**

New and improved antimicrobial countermeasures are urgently needed to counteract increased resistance to existing antimicrobial treatments and to combat currently untreatable or new emerging infectious diseases. We demonstrate that computational comparative genomics, together with experimental screening, can identify potential generic (i.e., conserved across multiple pathogen species) and novel virulence-associated genes that may serve as targets for broad-spectrum countermeasures.

**Results:**

Using phylogenetic profiles of protein clusters from completed microbial genome sequences, we identified seventeen protein candidates that are common to diverse human pathogens and absent or uncommon in non-pathogens. Mutants of 13 of these candidates were successfully generated in *Yersinia pseudotuberculosis *and the potential role of the proteins in virulence was assayed in an animal model. Six candidate proteins are suggested to be involved in the virulence of *Y. pseudotuberculosis*, none of which have previously been implicated in the virulence of *Y. pseudotuberculosis *and three have no record of involvement in the virulence of any bacteria.

**Conclusion:**

This work demonstrates a strategy for the identification of potential virulence factors that are conserved across a number of human pathogenic bacterial species, confirming the usefulness of this tool.

## Background

Microbial disease is the major cause of human death and morbidity and for many infectious diseases, no preventive vaccines are available [1]. Where therapies do exist, escalation of resistance to antimicrobials hinders treatment of common bacterial infections and accentuates the need for new approaches [2,3]. Therefore, it is imperative to identify appropriate targets for medical countermeasures such as antimicrobial drugs or cross-protective vaccines active against several pathogenic strains or species. An alternative to "killing" bacteria, which exacerbates the selection of antimicrobial resistance, is to "disarm" bacteria by interfering with their capacity to be virulent, thus enabling the bacterium to survive and evoke an appropriate immune protection [4]. Targeting such virulence factors through the development of antivirulence (as opposed to antimicrobial) compounds has indicated that it is possible to target common virulence genes [5,6].

Virulence is typically described as the damage a pathogen causes to the host during infection [7]. Gene products that contribute to virulence can therefore be described as virulence factors. Traditionally a gene has been classified to encode a virulence factor by experimentally introducing a mutation into the protein of interest and determining whether virulence of the resultant mutant is reduced. Genome-wide screens for identifying novel virulence factors have traditionally employed transposon mutagenesis to inactivate genes in a selected bacterial strain and then screening the resulting insertion mutants for attenuation in an appropriate animal infection model. An adaptation of this method has been the incorporation of unique DNA tags in signature-tagged mutagenesis (STM) which enables mutants to be screened *en masse *in animal infection models [8]. However, this approach is limited to a single strain of a particular bacterial species and a particular infection model. Therefore the approach typically identifies highly specific virulence factors with limited extrapolation to generic virulence determinants of other pathogens. Another approach is to identify genes up-regulated *in vivo*, such as *in vivo *expression technology (IVET) [9]. However, this approach also identifies genes other than those required for virulence and both STM and IVET identify genes in a pathogen that are also present in non-pathogens.

Computational approaches to identifying virulence factors have often been made through whole-genome comparisons of two or more bacteria, where the presence or absence of genes between closely related pathogenic and non-pathogenic strains can suggest genes that potentially play a role in virulence [10,11]. For example, Garbom et al. [12] identified novel virulence-associated genes in *Yersinia pseudotuberculosis *by looking at the hypothetical genes (genes of unknown function) conserved in six human microbial pathogens. Expanding on this work, we have used whole proteome searches to identify virulence-associated proteins common to diverse pathogenic bacteria that are absent in non-pathogenic species. The identified factors can then be exploited for the development of medical countermeasures such as antimicrobials, vaccines or diagnostics.

In contrast to computational approaches based on similarity, genomic context methods involve several non-similarity based approaches to predict protein functions and interactions [13-17]. Phylogenetic profiles particularly suit our objective of identifying conserved virulence factors across multiple human pathogenic species. The method was originally designed to identify functionally-related proteins that evolve in a correlated fashion by characterizing proteins by a binary string that encodes the presence or absence of the protein in every known genome [18]. The method has been improved and expanded in numerous ways, including new approaches to characterize profile patterns by domains [19] and protein families [20] and by integrating phylogenetic information to compute probabilities of observing different profile strings [21,22]. Phylogenetic profiles have been used to identify virulence factors related to bacterial food poisoning [23] and intracellular pathogenesis [24]. In this work, we have utilized a similar approach to identify potential virulence factors present in a group of extreme human pathogens, bacteria from the Centers for Disease Control category A and B pathogen lists (). We impose two primary criteria: first, the putative target genes must be broadly present in the diverse pathogens within these two groups. Second, the putative target genes should be absent or highly divergent in non-pathogens. This enhances the likelihood that candidates are implicated in virulence and minimizes the potential activity of future countermeasures against the host commensal flora.

## Results

### Clustering of proteins

Our targets of interest are proteins that are present in diverse pathogens but absent from non-pathogens, and therefore we had to decide which proteins in different organisms should be treated as the same protein. We began by doing an all against all BLAST comparison of a collection of 617,000 proteins from all 214 completely sequenced microbial genomes available at the time. The resulting 123 million BLAST hits were grouped into clusters using single-linkage clustering at eight different percent identity and query coverage cut-offs. Finally, phylogenetic profile tables were created to summarize the presence or absence of proteins within a cluster across all genomes.

### Searching for virulence-associated proteins

We searched for virulence-associated proteins in the phylogenetic profile tables using the BLAST Clusters link on the Toxin and Virulence Factor website (TVFac) at Los Alamos National Laboratory (). We assigned representative strains taken from the CDC category A and B pathogen lists to the target group (Table 1). Fifty-two non-pathogenic strains were included in the background group (Additional file 1). These were selected from the 214 genomes as having no association with human pathogenesis, or in fact no pathogenesis in animal and plants. Bacteria that have been identified as opportunistic pathogens were excluded from both lists as their mode of action may be as much to do with an immunocompromised host as the possession of traditional virulence factors by the bacteria. Next, we selected a minimum of five hits to the category A and B pathogens and maximum of three hits to non-pathogens and identified 1024 potential generic virulence factor candidates using the 50% identity and 90% coverage cutoffs (Table 2). Other combinations were exhaustively tested but more stringent cutoffs resulted in too few candidates whilst less stringent ones gave too many for our purposes.

**Table 1:** Pathogen strains in profile searches

**Phylum or Class**	**Strain**	**Abbreviation**
Firmicutes	*Bacillus anthracis *str. Ames Ancestor	Ba
Alphaproteobacteria	*Brucella melitensis *16M	Bme
Alphaproteobacteria	*Rickettsia prowazekii *str. Madrid E	Rp
Betaproteobacteria	*Burkholderia mallei *ATCC 23344	Bma
Betaproteobacteria	*Burkholderia pseudomallei *K96243	Bp
Gammaproteobacteria	*Coxiella burnetii *RSA 493	Cb
Gammaproteobacteria	*Escherichia coli *O157:H7	Ec
Gammaproteobacteria	*Francisella tularensis *subsp. tularensis Schu 4	Ft
Gammaproteobacteria	*Salmonella enterica *serovar Typhi Ty2	Se
Gammaproteobacteria	*Salmonella typhimurium *LT2	St
Gammaproteobacteria	*Shigella flexneri *2a str. 301	Sf
Gammaproteobacteria	*Vibrio cholerae *O1 biovar eltor str. N16961	Vc
Gammaproteobacteria	*Yersinia pestis *CO92	Yp
Gammaproteobacteria	*Yersinia pseudotuberuculosis *IP32953	Yptb

Strains of select agents (CDC category A and B bacteria) assigned to the target group in profile searches. Genomes sequences from some select agents were unavailable at the time (*Clostridium botulinum *and *Chlamydia psittaci*) and the toxin-producing strains of *Staphylococcus aureus *and *Clostridium perfringens *were excluded from the searches. Where multiple strains had been sequenced (such as for *Bacillus anthracis *and *Yersinia pestis*), a single representative strain was selected so that results were not skewed to favor species with multiple entries.

**Table 2:** Summary of candidates from profile searches.

		**Non-pathogens**			
		**0**	**1**	**2**	**3**
Pathogens	5	368	125	44	33
	6	20	119	145	82
	7	9	17	13	20
	8	1	4	4	14
	9	1	0	2	3

A total of 1024 potential virulence factor clusters were identified in the phylogenetic profile tables. At least five hits to pathogens of interest were required and no more than three hits to non-pathogens were allowed.

### Selecting candidates for experimental screening

Since it was impractical to experimentally test all 1024 candidates, additional down-selection was performed to narrow the number of candidates to a more manageable number. The final proteins used in experimental testing were selected based on a number of different criteria. The selected protein is required to have an ortholog in *Y. pseudotuberculosis *since this was our experimental pathogen to investigate virulence and 87 candidates were found to have no *Y. pseudotuberculosis *homolog in the cluster. Since it is possible that one closely-related protein could compensate for the loss in function of the other [25], a further 21 clusters with multiple hits to *Y. pseudotuberculosis *were also removed. Since only completely sequenced microbial genomes were included in the database, each target protein was also compared against protein sequences from eukaryotes and other organisms by following links on TVFac to pre-computed BLAST results available at NCBI (National Center for Biotechnology Information). Those targets with an identity of 50% or more to eukaryotes, and/or 50% or greater identity to 3 or more non-pathogens, were also removed from the list. By these considerations the number of candidates was narrowed down to 17 potential targets for experimental testing, of which thirteen of these were successfully constructed as mutants (Table 3).

**Table 3:** Selected targets

**CI**	**YPTB Locus**	**Definition**	**50-90 Cluster ID**	**Total non-pathogens**	**Total pathogens**	**Pathogen strains**
**0.01**	2410	Magnesium transport ATPase mgtB	1035	3	8	Bme, Bma, Bp
**0.03**	2913	Deoxyribopyrimidine photolyase phrB	2801	0	6	Vc
**0.06**	2705	Putative manganese transport protein mntH	1611	3	8	Ba, Bma, Bp
**0.12**	3827	Outer membrane biogenesis protein	2798	0	5	-
**0.13**	0181	Putative protohaeme IX biogenesis protein hemY	3642	0	5	-
**0.16**	0756	Superoxide dismutase C sodC	1306	0	9	Bme, Cb, Ft, Vc
0.39	1340	Lysine specific permease cadR	563	2	9	Ba, Bma, Bp, Ft
0.42	0242	Glycerophosphoryl diester phosphodiesterase glpQ	2411	0	7	Bma, Bp
0.45	2699	Hypothetical protein	3924	0	7	Bma, Bp
0.76	1424	Hypothetical protein	1031	3	9	Bma, Bp, Ft, Vc
0.87	3166	Thiol:disulphide interchange protein dscB	3283	0	5	-
2.38	0188	Frataxin-like protein cyaY	2139	2	6	Vc
3.15	3505	Stringent starvation protein sspB	1207	3	7	Cb, Vc
ND	1167	Phosphate starvation protein	3911	0	6	-
ND	1251	Ecotin	3380	0	6	-
ND	2026	MviN-like protein	1296	3	9	Bma, Bp, Cb
ND	2995	Putative surface antigen	2296	2	7	Vc

Virulence-associated genes identified by computational methods and selected for experimental screening. These targets have been ranked by their competitive index (CI) value in *Y. pseudotuberculosis*. The CI value shown is the mean of the CI calculated from 3 spleens individually plated in triplicate. The top six candidates are deemed virulence-related by our experimental criteria. Hits to pathogens include all five members of the Enterobacteriaceae (Ec, Se, Sf, St, Yp) plus the additional strains listed using abbreviations from Table 1.

The top hit from the cluster search was a superoxide dismutase C (SodC) with hits to nine category A and B pathogens and none from the non-pathogen group. Although several forms of this enzyme are found in both pathogenic and non-pathogenic bacteria as well as most other organisms including eukaryotes, this particular cluster included only pathogenic bacteria. This suggests that SodC from pathogens is distinct from non-pathogens at the amino acid sequence level when compared at the described cut-offs (50% identity and 90% coverage). Similarly, where targets are given as absent in a certain bacterium, this does not mean that no homolog can be found in that organism, but rather no homolog can be found at the cut-off levels used for this search.

### Testing virulence in *Y. pseudotuberculosis*

The growth rate of each mutant was compared to that of the wild-type (Additional file 2). No statistically significant differences between growth curves were observed (P > 0.05, using an ANCOVA, comparing sigmoidal dose models, fitted to the data). It is therefore unlikely that any of the genetic mutations in this study have affected the ability for them to grow in media. Mice were inoculated as described with doses ranging from 1 to 10^4 ^cfu of wild type *Y. pseudotuberculosis *strain IP32953 (see Methods for complete details). The median lethal dose (MLD) via the i.v. route was calculated to be 2 cfu, consistent with that previously reported [26]. Thirteen of the 17 target genes were inactivated by allelic replacement in *Y. pseudotuberculosis *and tested for reduced competitiveness with the wild type strain after i.v. challenge of mice. Four genes could not be constructed despite repeated attempts. For this study, a mutant was considered to be attenuated if it had a competitive index (CI) value of 0.2 or less. Based on this, six genes were identified that reduced virulence of *Y. pseudotuberculosis *(Table 3). None of the encoded proteins have been previously reported to play a role in virulence of *Y. pseudotuberculosis*, although three have been reported as playing a role in the virulence of other bacteria. It is possible however, that the genes themselves are not directly involved in virulence but have an effect on other genes that are.

## Discussion

The lifestyles of all of the pathogens selected for this study are known to involve survival in phagocytes and of the genes down-selected, several are implicated in the survival of bacteria within phagocytic cells. SodC is known to be a virulence factor in several pathogenic bacteria including *Neisseria meningitides *[27], *Burkholderia cenocepacia *[28], *Salmonella enterica *serovar *Typhimurium *and *Salmonella choleraesuis *[29] and *Brucella abortus *[30], and disruption of the *sodC *gene in these bacteria have generated attenuated mutants. Further work has characterized the role of superoxide dismutase C in the virulence in *Y. pseudotuberculosis *[31]. SodC is known to protect bacteria in phagosomes from the bactericidal action of superoxide anion, and SodC orthologs were found in most of the pathogens targeted for this study [32]. *Burkholderia mallei*, *Burkholderia pseudomallei *and *Bacillus anthracis *are also known to have SodC, but these enzymes do not show significant sequence homology with the cluster of SodC orthologs in this study at the 50% identity and 90% coverage cut-offs used.

Both Mg^2+ ^and Mn^2+ ^are believed to be limiting in the phagosome, and transport systems for these ions are common to many of the pathogens targeted for this study [33]. Bacterial MntH proteins are homologous to the eukaryotic NRAMP (natural-resistance-associated macrophage protein) family of proteins that transport either Mn^2+ ^or Fe^2+^. Mutants of the *mntH *gene (manganese transport protein) have been shown to be attenuated in *Salmonella typhimurium *[34]. In *S. typhimurium*, the magnesium transport ATPase gene, *mgtB*, is found on a pathogenicity island, SPI-3, and its expression is controlled by the PhoP/Q signal transduction system which is an essential system in *Salmonella *virulence [35].

HemY is a putative protoporphyrinogen IX oxidase that is found as part of the haem biosynthetic pathway in bacteria. Haem is a tetrapyrrole derivative commonly used as a prosthetic group in proteins such as cytochromes, catalases and peroxidases and is essential for respiration and defence against oxygen intermediates. This later function suggests a possible involvement as a host defense mechanism. Although no *hemY *gene has been associated with virulence to date, *Staphylococcus aureus *mutants of *hemB *have been shown to reduce virulence in the *Caenorhabditis elegans *infection model [36].

YPTB3827 is an uncharacterized protein which contains the COG domain COG2982, involved in outer membrane biogenesis [Cell envelope biogenesis, outer membrane]. The outer membrane of Gram-negative bacteria is made up of four major components: lipopolysaccharide, phospholipids, beta-barrel proteins, and lipoproteins [37]. Together they play a number of roles including maintaining the integrity of the cell, uptake and secretion of solutes and interaction with the host cell. YPTB3827 has not previously been associated with virulence. However, it is possible that disruption of this protein may affect virulence though interactions with the host cell, or through secretion of certain proteins. Further work on this protein will be required to further characterize its role and to elucidate its role in virulence.

Of the 17 targets, four could not be constructed as mutants suggesting that their function may be essential to the virulence of the cell. Each of these were compared to the Database of Essential Genes (DEG: ) and two, YPTB2995 and YPTB2026, showed over 50% identity to proteins described as essential in this database [38].

## Conclusion

Of the 13 mutants constructed, six were identified as having potential association with the virulence of *Y. pseudotuberculosis *(42%), suggesting that this selection process - guided primarily by the comparative presence or absence of potential targets in pathogens versus non-pathogens - is a promising tool for the identification of potential virulence-associated proteins. However, further work is needed to confirm whether these targets are important to *Y. pseudotuberculosis *outside the laboratory and whether these targets are also associated with virulence in other pathogens. Work is currently underway to characterize each of the targets and to demonstrate their roles in virulence. This work has already been completed for superoxide dismutase where it has been shown to be essential for the virulence of *Y. pseudotuberculosis *in both insect and mammalian hosts [31]. Similarly, to confirm the identified targets as generic, we are now carrying out studies in a range of bacterial pathogens.

An advantage of our approach is that commensal flora, which often play important roles in the well-being of humans, should be minimally affected. This is dramatically illustrated in the development of *Clostridium difficile*-associated colitis where the administration of broad-spectrum antibiotics significantly impacts the commensal gut flora producing an environment where the pathogenic *C. difficile *can proliferate [39]. Additional grounds for targeting virulence per se is furnished by recent metagenomic studies in humans, which suggest that the human metagenome contains several orders of magnitude more microbial genes than *Homo sapiens *genes and that our bodies themselves contain perhaps ten times as many microbial cells as "human" ones [40,41]. Avoiding potential disruption to this vast microbial community is thus highly desirable as commensals are now known to contribute to gut immunity and the synthesis of vital nutrients [39,42,43].

Since this work was started, the number of whole genome sequences has increased greatly and new protein family and cluster databases are readily available for searching. The TVFac website now includes phylogenetic profile tables created from 14 database cross-references and three UniRef clusters in UniProt [44]. As of July 2009, over 1430 microbial and nearly 80 eukaryotic genomes are currently represented in these tables. Further studies could be carried out with these extended datasets with the possibility of identifying more potential candidates. The success rate we observed in spite of these limitations suggests that improved methods along the lines suggested should further enhance the discovery of virulence-associated proteins that are conserved within a number of human pathogenic bacteria.

## Methods

Our approach in finding virulence-associated genes common to human pathogens followed three levels of computational pre-screening followed by experimental analysis of the resulting candidates. The computational steps reduced the number of potential targets from over 77,500 to 17 candidates. The experimental screening suggests that six of these candidates are virulence factors in *Y. pseudotuberculosis *and potential targets for countermeasures (flowchart in additional file 3).

### Computational methods

A collection of 617,000 proteins was downloaded from 214 completely sequenced microbial genomes available on the NCBI ftp site () in February 2005. The entire set of proteins was blasted all against all using BLASTP 2.2.10 on a 240-node Linux cluster. Soft-masking was used to avoid initial matches based on low complexity sequences and to allow extensions through masked regions. For each query protein, the top 1000 subject proteins with an e-value below 10^-5 ^were retained producing a final tab-delimited blast output file with almost 123 million rows of data. The proteins were grouped into clusters at eight different percent identity and query coverage cutoffs using a custom-built single-linkage clustering program written in C++. The clustering results were loaded into a MySQL database and cross-tabulations were run to create profile tables summarizing the presence or absence of proteins across all genomes. For example, 418,000 proteins were grouped into 77,500 clusters ranging in size from 2 to 506 proteins using 50% identity and 90% query coverage cut-offs. The corresponding profile table therefore contains 77,500 rows for each cluster and 214 columns, one for each genome.

The profiles tables were searched using the BLAST Clusters link on Toxin and Virulence Factor website (). In order to facilitate comparisons among groups of organisms in the profile tables, we first collected detailed information on host-pathogen interactions including the source of infection, methods of transmission and the ability of the pathogen to cause infection. The organism-based annotations were then linked to the search page to allow users to quickly highlight and select a target and background group. We selected "CDC pathogens -species" to highlight one representative strain of category A and B pathogens and moved those 13 taxa to the target group (Table 1). Next, we highlighted "non-pathogen Bacteria" and moved those 52 taxa to the background group. Finally, we selected a minimum of five hits to the target and 3 hits to the background and the 50% identity and 90% query coverage cutoffs. These search criteria identified 1024 cluster profiles summarized in Table 2.

### Experimental methods

Bacteria and plasmids used in this work are listed in additional file 4. *Y. pseudotuberculosis *strain IP32953 was selected because the complete genome sequence is available [45]. It was maintained in Luria-Bertani (LB) broth and on LB agar. Plasmid pAJD434 [46] and PCR products were introduced into *Y. pseudotuberculosis *IP32953 by electroporation. PCR products were purified using Millipore Microcon Ultracel YM-100. All *Y. pseudotuberculosis *IP32953 strains containing the pAJD434 plasmid were grown in LB media supplemented with 100 μg/ml trimethoprim, and 0.8% arabinose when λ Red Recombinase genes were required to be induced at 28°C. Mutants constructed in *Y. pseudotuberculosis *IP32953 were grown in LB supplemented with kanamycin at a final concentration of 50 μg/ml. Congo-red magnesium oxalate (CR-MOX) agar were prepared as described by Riley and Toma [47]. Unless otherwise stated, chemicals were purchased from Sigma-Aldrich (Poole, United Kingdom). Enzymes were purchased from Promega Ltd (Southampton, United Kingdom).

Construction of *Y. pseudotuberculosis *mutants was carried out using a previously published method [48]. Briefly, primers were designed for each target gene to be disrupted that included 20 bp complementary to the 5' or 3' sequence of the kanamycin gene of the plasmids pK2 or pUC4K followed by 50 bp of upstream or downstream sequence flanking the gene to be disrupted. PCR products were generated using the plasmid pK2 as a template, excess template was digested with *DpnI *and the PCR products were transformed into *Y. pseudotuberculosis *IP32953 pAJD434, by electroporation. Following overnight incubation at 28°C in LB supplemented with 0.8% arabinose, transformants were selected on LB agar supplemented with kanamycin (50 μg/ml) and trimethoprim (100 μg/ml) for 48 h at 28°C. Transformants were screened by PCR using target gene-specific and kanamycin gene-specific primers (Additional file 5). Mutant strains were cured of the pAJD434 plasmid by growth at 37°C in LB media supplemented with kanamycin (50 μg/ml). Cured mutant strains were screened for the virulence plasmid pYV by PCR for two genes located on this plasmid; *virF *and *yscC *(Additional file 6). The retention of the *Yersinia *virulence plasmid (pYV) was also confirmed by culture on CR-MOX plates, where plasmid retention results in small red colonies and plasmid loss results in large pink colonies [47].

To measure bacterial growth, mutants and wild-type bacteria were grown overnight in 20 ml LB broth (wild type) or 20 ml LB broth + kanamycin (50 μg/ml, mutants) with shaking at 28°C. 100 ml of fresh culture of the same medium were seeded from the over-night cultures and grown at 28°C as before. Growth was recorded over 24 hours by measuring the OD_600 _at various time-points and the growth curves were graphed and analysed using Graphpad PRISM v4.0. Data was fit to the model: Y = Bottom + (Top-Bottom)/(1+10^((LogEC50-X)*HillSlope)), where X is the logarithm of time, Y is the response; Y starts at the Bottom and goes to the Top with a sigmoid shape. Growth curves were compared using an ANCOVA (Analysis of Covariance).

The median lethal dose (MLD), the expected median dose required to produce morbidity or death in 50% of the population tested, was determined as previously described [49]. Briefly, groups of six female 6-week-old BALB/c mice (Charles River laboratories) were infected via intravenous (i.v.) injection with 0.1 ml serially diluted (in sterile PBS) exponential-phase cultures grown at 28°C in LB broth (wild type) or LB broth supplemented with kanamycin (mutant). Humane endpoints were strictly observed, and animals deemed incapable of survival (unable to right themselves or unresponsive to a pinch on the foot or tail) were killed by cervical dislocation. The MLD was calculated by the method of Reed and Muench [50].

For *in vivo *competitive index studies, mutant and wild-type strains were grown separately to exponential phase in 20 ml LB broth with shaking. Broth cultures were then centrifuged (10 minutes, 4,000 g) and the pellet re-suspended in 10 ml sterile PBS and centrifuged again (10 minutes, 4,000 g). The bacteria were washed and re-suspended in 10 ml PBS and the optical density adjusted to an OD_600 _of 0.55 to 0.6 with sterile PBS. Wild type and mutant bacterial suspensions were then mixed in a 1:1 ratio and serially diluted with sterile PBS to give an inoculation concentration of approximately 1 × 10^3 ^cfu/ml. Groups of 6 mice were then dosed with 0.1 ml of this solution by the i.v. route as above. Retrospective viable counts were determined by plating out dilutions (in triplicate) on LB agar and LB agar supplemented with kanamycin to determine the input ratio. After 5 days, spleens were recovered and passed through sieves (70 μm; Becton Dickinson) to produce a cell suspension in 3 ml of PBS. Cell suspensions were serially diluted in sterile PBS and plated onto LB agar and LB agar supplemented with kanamycin to determine the output ratio. The competitive index (CI) is defined as the output ratio (mutant/wild type) divided by the input ratio (mutant/wild type) [51,52].

## Authors' contributions

CJS designed the computational tools and MLD conducted the down-selection and both authors drafted and revised manuscripts. JDG, JS, BL assisted with computational steps. IAC, DCF, AVK and AR assisted with experiments. CJS, MLD, PCO, BWW, RWT and MW designed the research. All authors read and approved the final manuscript.

## Supplementary Material

Additional file 1 **Non-pathogen species in profile searches**. Species assigned to non-pathogen group in profile searches. The numbers of strains (if different than one) are listed in parentheses after the species name. The habitat and temperature range are taken from the NCBI organism information table . Click here for file

Additional file 2 **Growth curves**. Growth curve for wild type and mutants over 24 hours measured by optical density (600 nm). Curve shown is a sigmoidal dose response model that best describes the data in its integrity; no significant differences were seen between individual models (p > 0.05 using an ANCOVA, comparing sigmoidal dose models, fitted to the data). Plot symbols in the key correspond to *Y. pseudotuberculosis *locus tags listed in Table 3. Click here for file

Additional file 3 **Overview of methodology**. Each of the first three computational (or semi-computational) steps results in a large reduction of the potential search space (which can not be usefully shown to scale). Clustering provides an operational definition of the "same" protein in different organisms. Profiling determines which clusters are overrepresented in pathogens. Filtering selects those clusters which meet experimental criteria. The resulting candidates are then screened experimentally. Click here for file

Additional file 4 **Bacterial strains and plasmids**. Characteristics and source of bacterial plasmids used in experimental work. Click here for file

Additional file 5 **Mutagenesis primers**. A list of all primers used in the mutagenesis of *Y. pseudotuberucosis* Click here for file

Additional file 6 **Screening primers**. A list of all primers used for screening and confirmation of this work Click here for file
