# Supplementary material for: Steps toward broad-spectrum therapeutics: discovering virulence-associated genes present in diverse human pathogens
Source: BMC Genomics. 2009 Oct 29;10:501. doi: 10.1186/1471-2164-10-501 (PMC2774872; doi:10.1186/1471-2164-10-501)
Supplement: Additional file 1 — Non-pathogen species in profile searches. Species assigned to non-pathogen group in profile searches. The numbers of strains (if different than one) are listed in parentheses after the species name. The habitat and temperature range are taken from the NCBI organism information table . [file 1471-2164-10-501-S1.doc]

Additional file 1. Non-pathogen species in profile searches

| Group | Species | Habitat | Temperature range |
| --- | --- | --- | --- |
| Actinobacteria | Bifidobacterium longum | Host-associated | Mesophilic |
| Actinobacteria | Corynebacterium efficiens | Multiple | Mesophilic |
| Actinobacteria | Corynebacterium glutamicum | Multiple | Mesophilic |
| Actinobacteria | Symbiobacterium thermophilum | Terrestrial | Thermophilic |
| Aquificae | Aquifex aeolicus | Specialized | Hyperthermophilic |
| Bacteroidetes/Chlorobi group | Chlorobium tepidum | Specialized | Thermophilic |
| Chloroflexi | Dehalococcoides ethenogenes | Multiple | Mesophilic |
| Cyanobacteria | Gloeobacter violaceus | Terrestrial | Mesophilic |
| Cyanobacteria | Nostoc sp. | Multiple | Mesophilic |
| Cyanobacteria | Prochlorococcus marinus (2) | Aquatic | Mesophilic |
| Cyanobacteria | Synechococcus elongatus | Aquatic | Mesophilic |
| Cyanobacteria | Synechococcus sp. | Aquatic | Mesophilic |
| Cyanobacteria | Synechocystis sp. | Aquatic | Mesophilic |
| Cyanobacteria | Thermosynechococcus elongatus | Specialized | Thermophilic |
| Deinococcus-Thermus | Deinococcus radiodurans | Terrestrial | Mesophilic |
| Deinococcus-Thermus | Thermus thermophilus (2) | Specialized | Thermophilic |
| Firmicutes | Bacillus halodurans | Multiple | Mesophilic |
| Firmicutes | Bacillus subtilis | Terrestrial | Mesophilic |
| Firmicutes | Clostridium acetobutylicum | Multiple | Mesophilic |
| Firmicutes | Geobacillus kaustophilus | Aquatic | Thermophilic |
| Firmicutes | Lactobacillus johnsonii | Host-associated | Mesophilic |
| Firmicutes | Listeria innocua | Multiple | Mesophilic |
| Firmicutes | Oceanobacillus iheyensis | Multiple | Mesophilic |
| Firmicutes | Streptococcus thermophilus (2) | Multiple | Thermophilic |
| Firmicutes | Thermoanaerobacter tengcongensis | Specialized | Hyperthermophilic |
| Planctomycetes | Rhodopirellula baltica | Aquatic | Mesophilic |
| Alphaproteobacteria | Bradyrhizobium japonicum | Host-associated | Mesophilic |
| Alphaproteobacteria | Caulobacter crescentus | Aquatic | Mesophilic |
| Alphaproteobacteria | Mesorhizobium loti | Multiple | Mesophilic |
| Alphaproteobacteria | Rhodopseudomonas palustris | Multiple | Mesophilic |
| Alphaproteobacteria | Silicibacter pomeroyi | Aquatic |  |
| Alphaproteobacteria | Sinorhizobium meliloti | Multiple | Mesophilic |
| Alphaproteobacteria | Zymomonas mobilis | Multiple | Mesophilic |
| Betaproteobacteria | Azoarcus sp. | Terrestrial | Mesophilic |
| Betaproteobacteria | Nitrosomonas europaea | Multiple | Mesophilic |
| delta/epsilonsubdivisions | Desulfotalea psychrophila | Specialized | Psychrophilic |
| delta/epsilonsubdivisions | Geobacter sulfurreducens | Multiple | Mesophilic |
| delta/epsilonsubdivisions | Wolinella succinogenes | Host-associated | Mesophilic |
| Gammaproteobacteria | Buchnera aphidicola (3) | Host-associated | Mesophilic |
| Gammaproteobacteria | Candidatus Blochmannia | Specialized | Mesophilic |
| Gammaproteobacteria | Idiomarina loihiensis | Specialized | Mesophilic |
| Gammaproteobacteria | Mannheimia succiniciproducens | Host-associated | Mesophilic |
| Gammaproteobacteria | Methylococcus capsulatus | Multiple | Thermophilic |
| Gammaproteobacteria | Photobacterium profundum | Multiple | Psychrophilic |
| Gammaproteobacteria | Wigglesworthia endosymbiont | Host-associated | Mesophilic |
| Thermotogae | Thermotoga maritima | Specialized | Hyperthermophilic |
