# Supplementary material for: Steps toward broad-spectrum therapeutics: discovering virulence-associated genes present in diverse human pathogens
Source: BMC Genomics. 2009 Oct 29;10:501. doi: 10.1186/1471-2164-10-501 (PMC2774872; doi:10.1186/1471-2164-10-501)
Supplement: Additional file 2 — Growth curves. Growth curve for wild type and mutants over 24 hours measured by optical density (600 nm). Curve shown is a sigmoidal dose response model that best describes the data in its integrity; no significant differences were seen between individual models (p > 0.05 using an ANCOVA, comparing sigmoidal dose models, fitted to the data). Plot symbols in the key correspond to Y. pseudotuberculosis locus tags listed in Table 3. [file 1471-2164-10-501-S2.doc]

Additional file 2. Growth curves
