# Supplementary figures and images for: Steps toward broad-spectrum therapeutics: discovering virulence-associated genes present in diverse human pathogens
Source: BMC Genomics. 2009 Oct 29;10:501. doi: 10.1186/1471-2164-10-501 (PMC2774872; doi:10.1186/1471-2164-10-501)

Additional file 3. Overview of methodology

***
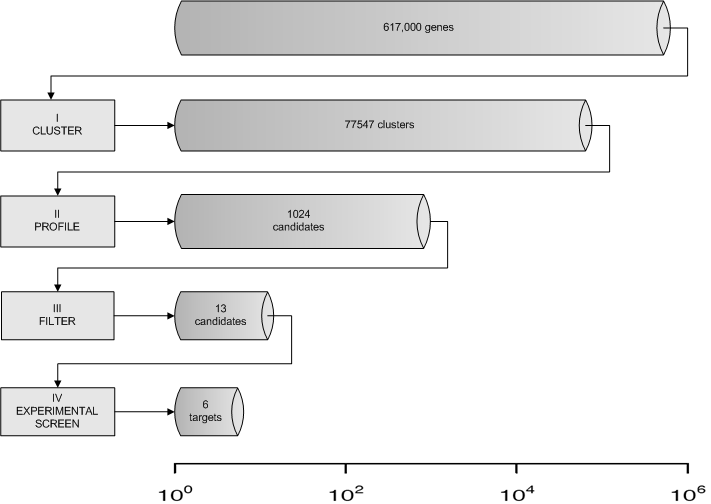
***

Supplement: Additional file 3 — Overview of methodology. Each of the first three computational (or semi-computational) steps results in a large reduction of the potential search space (which can not be usefully shown to scale). Clustering provides an operational definition of the "same" protein in different organisms. Profiling determines which clusters are overrepresented in pathogens. Filtering selects those clusters which meet experimental criteria. The resulting candidates are then screened experimentally. [file 1471-2164-10-501-S3.doc]
