# Supplementary material for: Steps toward broad-spectrum therapeutics: discovering virulence-associated genes present in diverse human pathogens
Source: BMC Genomics. 2009 Oct 29;10:501. doi: 10.1186/1471-2164-10-501 (PMC2774872; doi:10.1186/1471-2164-10-501)
Supplement: Additional file 4 — Bacterial strains and plasmids. Characteristics and source of bacterial plasmids used in experimental work. [file 1471-2164-10-501-S4.doc]

Additional file 4. Bacterial strains and plasmids

| Strain or plasmid | Characteristics | Reference |
| --- | --- | --- |
| *Y. pseudotuberculosis* strain IP32953 |  | Gift from E. Carneil |
| pGEM®-T-Easy | Cloning vector | Invitrogen |
| pK2 | High copy number pGEM®-T-Easy cloning vector with a kanamycin cassette at the *Bgl* II restriction site | Taylor et al (2005). Microbiology 151: 1919-1926 |
| pUC4K | Cloning vector | GE Healthcare |
| pAJD434 | Encodes λ red recombinase genes under the control of an arabinose inducible promoter | Gift from A. Darwin |
