# Supplementary material for: Steps toward broad-spectrum therapeutics: discovering virulence-associated genes present in diverse human pathogens
Source: BMC Genomics. 2009 Oct 29;10:501. doi: 10.1186/1471-2164-10-501 (PMC2774872; doi:10.1186/1471-2164-10-501)
Supplement: Additional file 5 — Mutagenesis primers. A list of all primers used in the mutagenesis of Y. pseudotuberucosis [file 1471-2164-10-501-S5.doc]

Additional file 5. Mutagenesis primers

| Mutagenesis Primer | Primer Sequence 5’→3’ |
| --- | --- |
| YPTB 0181 H1 | TTCCTTGTTTTAACTGTGGGTATCGTGCTTGGCCCAATGTTGGCCGGACA CACAGGAAACAGCTATGACC |
| YPTB 0181 H2 | TTTCCACCAGCGTTTCAGACCTTCGCTGCCTTCGTCCGCCATGCACTGAT CAAGTCAGCGTAATGCTCTGC |
| YPTB 0188 H1 | GCTCTCTATAATCGCAGGTGGATCATGAAAAGCAATAGGAATCGAATATGGATC TGCCACGTTGTGTCTC |
| YPTB 0188 H2 | GTGTTCCAATTGAATACCAACCGAGTTGCTCGGCTGGTATTGCGATTTTAGCTCTGCCAGTGTTACAACC |
| YPTB 0242 H1 | CACTGGCACCGGAAAATACACTGGCCGCAATTGATGTCGGTGCGCGCTAC |
| YPTB 0242 H2 | GACCTGTTTTTATTATGGCGGGTATTGAGGGTCTTTATAGGAGAAGGTCA |
| YPTB 0756 H1 | TGACAAGGTTAAACCAGACTTAATCAACATATAAGGGATATAACAATATGGATCTGCCACGTTGTGTCTC |
| YPTB 0756 H2 | CACCAAACTGGCTACGCTCGGGTAGCCAGTTTAACTGTATCAGACGGTTAGCTCTGCCAGTGTTACAACC |
| YPTB 1340 H1 | GCGCAGCAGGGTGCACAGCGCTTACGCCGGGAGCTTAAATCGCGGCATTTCACAGGAAACAGCTATGACC |
| YPTB 1340 H2 | GATGCTCTTCACTCTCATCACGCCATTTTGGGAATTCCATCTCTTGGTATCAAGTCAGCGTAATGCTCTGC |
| YPTB 1424 H1 | TATCCTCAAACACTTTCCTGCTGGCTAAACTCCATTTTGGAGGATGTATGGATCTGCCACGTTGTGTCTC |
| YPTB 1424 H2 | ACGGGCCTCGTTTTCCAACAAGACAGGGATACCGTCGCGTACCGGGTAAGGCTCTGCCAGTGTTACAACC |
| YPTB 2410 H1 | TATTGAGCGGATAAGCTTCGTGCGTTTTGAATTAAAAGAGAAGCGTTATGGATCTGCCACGTTGTGTCTC |
| YPTB 2410 H2 | CGGAATAGTGTCTTGAGATCAACCAGGCTATTTGAAACCTTGCCCCCTCAGCTCTGCCAGTGTTACAACC |
| YPTB 2699 H1 | GCACGCTGGCGGCCAGTAAACCTCAGTTTATGGCATTTACTGGTCAACGCAAGTCAGCGTAATGCTCTGC |
| YPTB 2699 H2 | AGCGCCACACAAATCGTCGTCAGCGACATCGTCCTGTAAGGCCCGCACACAGGAAGCTATGACC |
| YPTB 2705 H1 | GATACATCTCGTCGCCCATTAAGGAAGATTAAACTCTCCTTGATGGGGCC CACAGGAAACAGCTATGACC |
| YPTB 2705 H2 | CTGCAAGGTTATATAAGCCTCTTCGATTTCAGCAATGTTCTCATAACCAT CAAGTCAGCGTAATGCTCTGC |
| YPTB 2913 H1 | CCGCAATGATTTACGGGTCACCGATAATTTGGCGCTTCATGCGGCCTGTC CACAGGAAACAGCTATGACC |
| YPTB 2913 H2 | TTAGGGATCCTTGTGCTATCCCGCTTAGCGGCTTCAAATGCCGCGAGTGT CAAGTCAGCGTAATGCTCTGC |
| YPTB 3166 H1 | ATCATCCACGCGCTTAATCGCTGTATGGAAAAAGATAGGATTTATAAATG |
|  | GATCTGCCACGTTGTGTCTC |
| YPTB 3166 H2 | TCAACCACCCGCTTTTAGTGAAGCCTGATGCGCATTAAGCATCTGTAGCA |
|  | GCTCTGCCAGTGTTACAACC |
| YPTB 3505 H1 | ATGGAGATGTCGGATATGTCTCCGCGCCGCCCTTACTTGTTGCGC GCATTGATCTGCCACGTTGTGTCTC |
| YPTB 3505 H2 | AACGCCGCCCTGAAGAGATAGAGCCTGTATTTATCCGAACCGCGAAAATTGCTCTGCCAGTGTTACAACC |
| YPTB 3816 H1 | CCGTTCTCCCTGCCACCGTTTTCTGCTATTCGGCCTGAAGATATCGTGCC CACAGGAAACAGCTATGACC |
| YPTN 3816 H2 | GCAACATGGCATCTAACTGCGGTTCACGGCCACGGAAGCGTTTGAACAGT CAAGTCAGCGTAATGCTCTGC |
| YPTB 3827 H1 | AGAATACGTAGATTCACAGCGACTGTACGTAGAAGCATGGAGCTATTATGGATCTGCCACGTTGTGTCTC |
| YPTB 3827 H2 | TTATTTCTTTTGCTGCATTTTTCGCAGTAACGGTGCGCACTGATTCTCTTGCTCTGCCAGTGTTACAACC |
| KanF1 | GCCATATTCAACGGGAAACG / CACAGGAAACAGCTATGACC |
| KanR1 | AAACTCACCGAGGCAGTTCC / CAAGTCAGCGTAATGCTCTGC |
| LcrVfor | ACAACTGGCTCTGCTAGAAC |
| LcrVrev | TCACAATACGCCACGCTTAG |
| VirFfor | TTCCAGAGCGAGGAGTTCAG |
| VirFrev | ATCCAGCGGCGAAACAATAC |
