# Supplementary material for: Steps toward broad-spectrum therapeutics: discovering virulence-associated genes present in diverse human pathogens
Source: BMC Genomics. 2009 Oct 29;10:501. doi: 10.1186/1471-2164-10-501 (PMC2774872; doi:10.1186/1471-2164-10-501)
Supplement: Additional file 6 — Screening primers. A list of all primers used for screening and confirmation of this work [file 1471-2164-10-501-S6.doc]

Additional file 6. Screening primers

| Screening Primer | Primer sequence |
| --- | --- |
| YPTB 0181 for | ATGCTGCGAGTCTTGCTGTT |
| YPTB 0181 rev | CGTACTTTGCGGCTTTGATC |
| YPTB 0188 for | ACTCATGCGTCCAAGCTCTC |
| YPTB 0188 rev | AGTTGCTCGGCTGGTATTGC |
| YPTB 0242 for | CAGTGGCAGGCAGTACATTG |
| YPTB 0242 rev | ATTATGGCGGGTATTGAGGG |
| YPTB 0756 for | GCGGGAGTATGAAGCATAAG |
| YPTB 0756 rev | CCGATTATCGCAGATTACCC |
| YPTB 1340 for | ATGACTCAGCAAAATACTAAAATCC |
| YPTB 1340 rev | TCACCGACTTGTCGGTTTC |
| YPTB 1424 for | CTTTCCTGCTGGCTAAACTC |
| YPTB 1424 rev | TCCAACAAGACAGGGATACC |
| YPTB 2410 for | GGGATATTGAGCGGATAAGC |
| YPTB 2410 rev | CACGCCCATTCAGTGGTTCC |
| YPTB 2699 for | CGTCGTAATCTGGCCGAGG |
| YPTB 2699 rev | GATGCCAC GTTCAGAAGCTG |
| YPTB 2705 for | ATGCTTAATGGCCGTGCTGT |
| YPTB 2705 rev | AACAGTGGCCGCAGCATTAC |
| YPTB 2913 for | ATGTCTACGCATCTGGTCTGGTT |
| YPTB 2913 rev | TCAATAACATGAAGGCTCCTTGC |
| YPTB 3166 for | CGCGCTTAATCGCTGTATGG |
| YPTB 3166 rev | GCAGGTAAATGGCTGTCATC |
| YPTB3505 for | GTTCTTGGCTTCACTGACTG |
| YPTB3505 rev | CGCCCTGAAGAGATAGAGCC |
| YPTB3816 for | ATGACAAACCCGCTGTTGACT |
| YPTB3816 rev | TTAGCCCTTAATACCGTAATGAC |
| YPTB 3827 for | CAGCGACTGTACGTAGAAGC |
| YPTB 3827 rev | GCTGCAAGATCACACCTGTC |
